# High-throughput identification of reference genes for research and clinical RT-qPCR analysis of breast cancer samples

**DOI:** 10.1186/2043-9113-3-13

**Published:** 2013-07-22

**Authors:** Diana V Maltseva, Nadezda A Khaustova, Nikita N Fedotov, Elona O Matveeva, Alexey E Lebedev, Maxim U Shkurnikov, Vladimir V Galatenko, Udo Schumacher, Alexander G Tonevitsky

**Affiliations:** 1 SRC Bioclinicum, Moscow, Russia; 2 Faculty of Mechanics and Mathematics of Lomonosov Moscow State University, Moscow, Russia; 3 Institute of General Pathology and Pathophysiology, Moscow, Russia; 4 Department of Anatomy II: Experimental Morphology, University Cancer Center Hamburg, University Medical Center Hamburg-Eppendorf, Hamburg, Germany

**Keywords:** Reference genes, Microarrays, Reverse transcription quantitative real-time polymerase chain reaction (RT-qPCR), Gene expression, Breast cancer

## Abstract

**Background:**

Quantification and normalization of RT-qPCR data critically depends on the expression of so called reference genes. Our goal was to develop a strategy for the selection of reference genes that utilizes microarray data analysis and combines known approaches for gene stability evaluation and to select a set of appropriate reference genes for research and clinical analysis of breast samples with different receptor and cancer status using this strategy.

**Methods:**

A preliminary search of reference genes was based on high-throughput analysis of microarray datasets. The final selection and validation of the candidate genes were based on the RT-qPCR data analysis using several known methods for expression stability evaluation: comparative ∆Ct method, geNorm, NormFinder and Haller equivalence test.

**Results:**

A set of five reference genes was identified: *ACTB*, *RPS23*, *HUWE1*, *EEF1A1* and *SF3A1*. The initial selection was based on the analysis of publically available well-annotated microarray datasets containing different breast cancers and normal breast epithelium from breast cancer patients and epithelium from cancer-free patients. The final selection and validation were performed using RT-qPCR data from 39 breast cancer biopsy samples. Three genes from the final set were identified by the means of microarray analysis and were novel in the context of breast cancer assay. We showed that the selected set of reference genes is more stable in comparison not only with individual genes, but also with a system of reference genes used in commercial OncotypeDX test.

**Conclusion:**

A selection of reference genes for RT-qPCR can be efficiently performed by combining a preliminary search based on the high-throughput analysis of microarray datasets and final selection and validation based on the analysis of RT-qPCR data with a simultaneous examination of different expression stability measures. The identified set of reference genes proved to be less variable and thus potentially more efficient for research and clinical analysis of breast samples comparing to individual genes and the set of reference genes used in OncotypeDX assay.

## Background

Gene-expression analysis is ubiquitously used both in life sciences and in medical research [1-3]. Microarray and reverse transcription quantitative real-time polymerase chain reaction (RT-qPCR) analyses are commonly used to measure transcript abundance. Microarrays allow the massive parallel analysis of thousands of genes but still require significant time and financial expenses. RT-qPCR is used as a conventional routine method for expression profiling of a moderate number of genes and is highly suitable when only a small amount of sample is available [1,4]. High throughput thermal cyclers and PCR platforms help to overcome the limitation of RT-qPCR and to perform simultaneous expression analysis of tens and hundreds of genes for one or more samples depending on the platform [1].

In research practice these two technologies are able to solve a broad spectrum of questions supplementing each other. In clinical diagnostics RT-qPCR is still more popular with the advantage of speed and a relatively low cost [5].

Based on a totally different readout strategy, RT-qPCR has become the standard method for validation of microarray data. However, the application of RT-qPCR requires an appropriate normalization to obtain accurate and reliable quantification of gene expression levels. The purpose of normalization is to remove experimentally induced errors that can be introduced at a number of stages throughout the procedure (variability in sample acquisition and RNA extraction protocols, different reverse transcription reactions and PCR efficiencies) and to reveal the true biological changes in expression [4,6-8]. Lack of appropriate normalization can lead to misinterpretation of a target gene expression profile. This is especially pronounced when the samples come from different individuals, different tissues, different time courses, different environmental conditions, etc.

To date, the reference gene concept is a gold standard used for normalization [1]. According to this concept reference genes are endogenous controls whose expression remains stable across all sources of variation during the experimental workflow. Results of RT-qPCR studies that use improper reference genes (e.g. genes that are not constitutively expressed) can be significantly different from results obtained with proper reference genes [7,9,10]. So far, as ideal reference genes do not exist, it is essential to select appropriate reference genes for accurate normalization of the RT-qPCR results for each experimental condition. It is worth noting that the expression levels of many commonly used reference genes such as *GAPDH*, *ACTB*, *RPS18*, *UBC*, *B2M*, and *HPRT1* have been reported to vary considerably in multiple tissues and cells [4,7,9,11-15]. However, in the absence of a better choice, the majority of researchers continue to use these genes, sometimes even without preliminary validation [9,13,16]. Thus for many studies there is a necessity to identify other candidate reference genes. One of possible ways is RT-qPCR screening of all 535 transcripts involved in maintaining cellular function by RT-qPCR [11,15]. Another way in our genomic era is to use the wealth of accumulated available data obtained by high-throughput microarray and sequencing technologies. This idea is getting more and more popular and was used in some recent studies of plants [17-19], diatoms [20], mammals [21,22] and human [23-27]. Certain attempts were also made to assess the stability characteristics of transcripts in different tissue types and cells of several species in different physiological states [14,28,29]. However, no genes were identified as generally stably expressed. Thus it is still a problem to reveal genes that are stably expressed in a given biological context, and the analysis of well-annotated microarray data may be very helpful to solve this problem. This approach promises to be particularly fruitful for heterogenic samples like biopsies which may contain different cell types in different ratios that can vary widely between samples.

In this study, we identified a set of genes that can be used as a reference simultaneously for analysis of breast cancer samples with an unknown hormone receptor status and different cancer status. To achieve this goal we analyzed four available well-annotated Affymetrix Human Genome U133A array datasets containing different breast cancers and normal breast epithelium from breast cancer and cancer-free patients. Then we validated identified candidate reference genes by RT-qPCR on 39 fresh breast cancer biopsies and compared expression stability of these genes and commonly used control genes including *ACTB*, *GAPDH*, *RPLP0*, *GUSB* and *TFRC* that form a set of reference genes in a commercial Oncotype DX Breast Cancer diagnostic test [2].

In order to select an optimal set of reference genes we developed a new strategy that utilizes the advantages of the most widely used algorithms for gene stability evaluation: comparative ∆Ct method [8], geNorm [12], NormFinder [1,30] and Haller equivalence test [31]. Each of these popular methods for gene stability evaluation has its own advantages and pitfalls, and the developed strategy allows scientists to avoid weighing all the pros and cons of individual methods each time, but instead to combine all these methods. We showed that novel candidate reference genes identified by our microarray search have expression stability comparable to or even higher than the stability of commonly used reference genes. We also demonstrated that the selected set of 5 reference genes is more stable than the set of 5 reference genes used in Oncotype DX Breast Cancer diagnostic test. The RT-qPCR data used for the validation study was obtained in compliance with the global standardization accords presented in the MIQE guidelines [32], see Additional file 1.

## Methods

### Large-scale selection of an extended list of candidate reference genes using microarray data

Four GEO Series [33,34] of Affymetrix Human Genome U133A array data were taken: GSE17705 (title: “Endocrine Sensitivity Index Validation Dataset”, 298 samples, [35]), GSE10780 (title: “Proliferative genes dominate malignancy-risk gene signature in histologically-normal breast tissue”, 185 samples, [36]), GSE20711 (title: “Epigenetic portraits of human breast cancers (expression data)”, 90 samples, [37]), GSE20437 (title: “Histologically normal epithelium from breast cancer patients and cancer-free prophylactic mastectomy patients”, 42 samples, [38]).

For each data series raw microarray data were processed using Bioconductor [39] xps package [40] implementation of RMA [41]. For each probeset a variability characteristic was computed as a ratio of 95- and 5-percentiles of log-transformed expression levels, and an ascending ordering of probesets was performed according to this variability characteristic. After these steps four lists of probesets were obtained (each corresponding to one data series). Genes corresponding to probesets that belong to top-500 for all four lists were included into the extended list of candidate reference genes.

The variability characteristic used for stability ranking is methodologically close to the interquartile range, but controls expression variation for 90 percent of samples in a dataset instead of 50 percent. Unlike mean deviation and standard deviation that are commonly used for expression stability comparison [14,15,24] the ratio of quantiles is absolutely stable with respect to data outliers.

For genes with stable expression the stability ranking based on the ratio of 95- and 5-percentiles of log-transformed expression levels is similar to ranking based on a relative width of expression range in a logarithmic scale: if 5-percintile *p*_*5*_ of log-transformed expression level equals *m(1-w)*, and 95-percintile *p*_*95*_ equals *m(1 + w)*, and 0 ≤ *w*<< 1 then

$$\begin{array}{l} \frac{p_{95}}{p_{5}}=\frac{m\left(1+w\right)}{m\left(1-w\right)}=\left(1+w\right)\left(1+w+w^{2}+\ldots \right)\\ \;=1+2w+\frac{2w^{2}}{1-w}\approx 1+2w. \end{array}$$

The top-500 lists of most stable probesets for four datasets used for the generation of an extended list of candidate genes contained 347–410 unique gene symbols. However, pairwise intersection of top-500 lists corresponding to two datasets (GSE17705 – ER-positive breast cancer patients [35]; GSE20711 – breast cancer patients [37]) contained only 48 unique gene symbols, and interception of top-500 lists corresponding to three datasets (a small dataset GSE20437 – histologically normal epithelium from breast cancer patients and cancer-free prophylactic mastectomy patients [38] – added) contained 27 unique gene symbols. Hence, our extended list of candidate reference genes – it contained 25 unique gene symbols – was formed based mainly on three datasets out of four, and the forth dataset (GSE10780 – a larger set of histologically-normal breast tissues from breast carcinoma patients [36]) only validated our selection.

### Patients

For validation of reference genes, samples from 39 patients (mean age 60.5 years; range 35–87 years) were analyzed. Patients were treated for breast cancer at the Gertsen Moscow Research Institute of Oncology or the Moscow State Oncology Medical Center, Russia, in 2012. Patient selection was based only on the availability of tumor tissue. All patients gave written informed consent to access their tissues and review their medical records in accordance with the principles of the Declaration of Helsinki. All patients were treated by mastectomy. Patients did not receive neoadjuvant chemotherapy.

Histologically, 32 tumors were diagnosed as ductal carcinomas, 1 was a mucus-producing adenocarcinoma, and 6 were of other histological types. TMN classification of all tumors is presented in Additional file 2. Twenty five tumors (64%) were estrogen-receptor (ER) positive, and 14 cases (36%) were ER-negative. Progesterone receptors (PR) were detectable in 24 cases (61.5%), whereas 15 tumors (38.5%) were PR-negative. Detailed information about tumor receptor status is provided in Additional file 2. All tumor samples were placed in appropriate volume of RNAlater RNA stabilization reagent (Qiagen, Germany) within 30 min after mastectomy and incubated at 4°C at least overnight but no more than 3 days. If total RNA was not isolated from a tissue sample after 3 day incubation, the sample was frozen at −80°C.

### RNA extraction and quality control

Tumor samples stabilized in RNAlater reagent were crushed in liquid nitrogen. QIAzol lysis reagent (Qiagen, Germany) was added and the homogenate was centrifuged through a QIAshredder column (Qiagen, Germany). Total RNA was extracted from the eluate by the miRNeasy mini kit (Qiagen, Germany) according to the manufacturer’s instructions. Genomic DNA contamination was removed by performing DNaseI digestion on the RNA binding column for 15 min. Total RNA was eluted in 50 μl of RNase-free H_2_O and stored at −80°C. RNA yield and purity was assessed spectrophotometrically by measuring OD_260_ and OD_260/280_ ratio respectively in RNase-free H_2_O using NanoDrop ND-1000 (NanoDrop Technologies, USA). For all samples OD_260/280_ ratio was between 2.0 and 2.2.

RNA integrity was determined using the Experion Automated Electrophoresis Station with the standard-sensitivity RNA analysis kit according to the manufacturer’s instructions (Bio-Rad, USA). Values of RNA quality indicator (RQI) generated by Experion system’s software ranged from 6.4 to 9.4.

### Primer and probe design and validation

A gene list used for validation is presented in Table 1. Primer pairs and probes were designed using Primer3 software [42] according to the recommendations described in [43], and their specificity was determined with Primer-blast program [44]. All sequences were tested for potential secondary structure and dimerization formation using OligoAnalyzer 3.1 program [45]. Primer specificity was confirmed by agarose gel electrophoresis after RT-qPCR reaction. Validated sequences are shown in Table 2 (see details in Additional file 3). Oligonucleotides were synthesized by Syntol (Russia).

**Table 1:** Description of selected candidate reference genes

**Gene symbol**	**Gene name**	**GeneBank number**	**Gene function**	**Pseudo-genes**	Source***
*ACTB*	Beta actin	NM_001101.3	Cytoskeletal structural protein involved in are involved in cell motility, structure, and integrity.	No	Oncotype DX, [23,27,48]
*EEF1A1*	Elongation factor 1-alpha 1	NM_001402.5	Involved in protein biosynthesis.	No	Microarray search
*GAPDH*	Glyceraldehyde-3-phosphate dehydrogenase	NM_002046.4	Catalyzes the reversible oxidative phosphorylation of glyceraldehyde-3-phosphate.	Yes*	Oncotype DX, [23,48]
*GUSB*	Beta-D-glucuronidase	NM_000181.3	A hydrolase that degrades glycosaminoglycans.	Yes*	Oncotype DX, [48]
*HCFC1*	Host cell factor C1	NM_005334.2	Involved in control of the cell cycle.	No	Microarray search
*HUWE1*	E3 ubiquitin-protein ligase HUWE1	NM_031407.5	Involved in ubiquitination and subsequent proteasomal degradation of target proteins.	No	Microarray search
*MRPL19*	Mitochondrial ribosomal protein L19	NM_014763.3	Protein synthesis within the mitochondrion.	No	[23]
*PSMC4*	26S protease regulatory subunit 6B	NM_153001.2	Encodes a member of the triple-A family of ATPases that play a role in 26S proteasome assembly.	No	[23]
		NM_006503.3			
*PTMA*	Prothymosin alpha	NM_001099285.1 NM_002823.4	Prothymosin alpha may mediate immune function	No	Microarray search
*RPLP0*	Ribosomal protein P0	NM_001002.3	Encodes a ribosomal protein that is a component of the 60S subunit.	Yes*	Oncotype DX, [47]
		NM_053275.3			
*RPL23A*	Ribosomal protein L23a	NM_000984.5	Encodes a ribosomal protein that is a component of the 60S subunit.	Yes*	Microarray search
*RPL37A*	Ribosomal protein L37a	NM_000998.4	Encodes a ribosomal protein that is a component of the 60S subunit.	Yes*	Microarray search
*RPL39*	Ribosomal protein L39	NM_001000.3	Encodes a ribosomal protein that is a component of the 60S subunit.	No	Microarray search
*RPS23*	40S ribosomal protein S23	NM_001025.4	Encodes a ribosomal protein that is a component of the 40S subunit.	No	Microarray search
*SF3A1*	Splicing factor 3 subunit 1	NM_005877.4	Encodes subunit 1 of the splicing factor 3a protein complex. It is necessary for a formation of an active 17S particle that performs pre-mRNA splicing.	No	[23]
		NM_001005409.1			
*TBP*	TATA box binding protein	NM_001172085.1	General transcription factor playing a role in the activation of eukaryotic gene transcription.	No	[23,47]
		NM_003194.4			
*TFRC*	Transferrin receptor protein 1	NM_003234.2	Required for iron delivery from transferrin to cells.	No	Oncotype DX, [48]
		NM_001128148.1			
*TPT1*	Translationally-controlled tumor protein	NM_003295.2	Involved in calcium binding and microtubule stabilization.	No**	Microarray search, [23]

*Presented set of primer pair and probe do not anneal on these pseudogenes.

**The primers can anneal on TPT1-like protein gene with two mismatches, but the whole set has a unique specific probe.

***Oncotype DX - a commercial Oncotype DX Breast Cancer diagnostic test [2].

**Table 2:** Description of primers and probes for validated candidate reference genes

**Gene name**	Sequence of primers and probes*	**Amplicon size (bp)**	**E**
*HCFC1*	f-CCACATCGACTACACCACCAA	212	1.90
	r-CAGCTTCCTCTCACTGACCATC		
	pr-(FAM)-CGCCATCA(T-BHQ1)CTTCCGCATCGCC-(P)		
*TPT1*	f-ATCAGCCACGATGAGATGTTC	132	1.76
	r-ATTTCCACCAATGAGCGAGTC		
	pr-(FAM)-CGGACGGG(T-BHQ1)TGTGCCTGGAGGT-(P)		
*RPL37A*	f-AAGTCGGGATCGTCGGTAAA	112	1.97
	r-TTGCCACAGAAAGAGCAAGT		
	pr-(FAM)-TTGAAA(T-BHQ1)CAGCCAGCACGCCAAGT-(P)		
*PTMA*	f-ACCACCCAACCCAAACCA	238	1.85
	r-TGGTCACACCACAAGTAAAGT		
	pr-(FAM)-TCGGATGACCAAACCAGCC(T-BHQ1)TCGG-(P)		
*RPL23A*	f-CTGGAAGAGGCTGTGTATGAA	120	1.96
	r-TAGTAGATGGGTGTGTGAGGAC		
	pr-(FAM)-AGGGGAG(T-BHQ1)GTGGATTGGCTGGC-(P)		
*TBP*	f-TTCGGAGAGTTCTGGGATTGTA	227	1.83
	r-TGGACTGTTCTTCACTCTTGGC		
	pr-(FAM)-CCGTGGTTCG(T-BHQ1)GGCTCTCTTATCCTCAT-(P)		
*GUSB*	f-CTCATTTGGAATTTTGCCGATT	81	1.87
	r-CCGAGTGAAGATCCCCTTTTTA		
	pr-(FAM)-TGAACAG(T-BHQ1)CACCGACGAGAGTGCTGG-(P)		
*SF3A1*	f-AAGGGTCCAGTGTCCATCAAAGT	224	1.88
	r-GCCATGTTGTAGTAAGCCAGTGAG		
	pr-(FAM)-ACCAGGTC(T-BHQ1)CTGTCATTAAGGTGAAG-(P)		
*MRPL19*	f-TGTTTGCGGTTGTTAGTTCAC	239	1.87
	r-ACATTTCTGCTTGCCCTTCC		
	pr-(FAM)-CATTGCC(T-BHQ1)ACTGCTTACGATGAGTGC-(P)		
*ACTB*	f-CTGGAACGGTGAAGGTGACA	140	1.87
	r-AAGGGACTTCCTGTAACAACGCA		
	pr-(FAM)-TGGAGCGAGCA(T-BHQ1)CCCCCAAAGT-(P)		
*GAPDH*	f-GAAGGTGAAGGTCGGAGTC	226	1.99
	r-GAAGATGGTGATGGGATTTC		
	pr-(FAM)-CAAGCTTCCCG(T- BHQ1)TCTCAGCC-(P)		
*RPLP0*	f-CTGATCCATCTGCCTTTGTG	116	1.89
	r-GTCCGACTCCTCCGACTCTT		
	pr-(FAM)-AGCCCCAGC(T-BHQ1)AAGGTTGAAGCCA-(P)		
*TFRC*	f-AAAGGAAATGGGCCTGAGTTTA	92	1.96
	r-CATTCCCGAAATCTGTTGTTAG		
	pr-(FAM)-TGGCTGTATTC(T-BHQ1)GCTCGTGGAGA-(P)		
*PSMC4*	f-AGCTCTACAAGCAGATCG	80	1.95
	r-CAACATGGTCTTCCCACA		
	pr-(FAM)-AGGCGTCCTCATG(T-BHQ1)ATGGCCCACCT-(P)		
*HUWE1*	f- CAGAGTTGGACAGAGTGAAA	137	2.10
	r- TACACAGAGAGAGGAGGACA		
	pr-(FAM)-TCGTTCCATCTCCG(T-BHQ1)AAACCAG-(P)		
*EEF1A1*	f-TGAAAACTACCCCTAAAAGCCA	208	1.91
	r- TATCCAAGACCCAGGCATACT		
	pr- (FAM)-TAGATTCGGGCAAG(T-BHQ1)CCACCA-(P)		
*RPS23*	f- CGGTGCTTCTCTCTTTCGCT	110	2.00
	r- ATGCCACTTCTGGTCTCGT		
	pr- (FAM)-AGTGTCG(T-BHQ1)GGACTTCGTACTGC-(P)		
*RPL39*	f- TTATGCTGTCTGAAGGTCACGA	120	1.85
	r- AATCCAGCCAACCAACGTG		
	pr- (FAM)-TGGAGATT(T-BHQ1)CGACGTGTTTTCCTCTC-(P)		

*All oligodeoxynucleotide sequences are given in 5’ → 3’ direction, f – a forward primer, r – a reverse primer, pr – a probe.

The amplification efficiency of each set of primer pair and probe was determined by plotting the Ct values obtained for 7 serial dilutions of cDNA (1:25, 1:100, 1:250, 1:500, 1:1000, 1:2500, and 1:5000). For this aim cDNA was synthesized by reverse transcription of the RNA pool which was prepared by mixing of an equal amount of total RNA isolated from five random tumor samples from the biopsy collection. The corresponding RT-qPCR primer efficiencies (E) were calculated according to the equation E = 10^(−1/slope)^. Each reaction was performed in triplicates. All PCR-mixes were pipetted by the automated pipetting system epMotion 5075 (Eppendorf, Germany).

PCR efficiencies for all candidate reference genes were higher than 1.83 and lower than 2.1 (Table 2, see Additional files 4 and 5), except for *TPT1* (1.76). The amplicon sizes for all primer pairs are presented in Table 2.

### cDNA synthesis and quantitative RT-qPCR

RNA samples were reverse transcribed to cDNA with the Reverse Transcription reaction mix using random hexamer primer according to the manufacturer’s instruction (Syntol, Russia). Initially 2 μg of total RNA was incubated at 65°C for 5 min followed by an incubation with 10 μl 2.5× reaction mix, 1 μl of 15 OD/μl random hexamer, 1 μl of 50U/ μl MMLV reverse transcriptase, 1 μl of 5U/ μl RNase inhibitor and nuclease-free water in final volume 25 μl for 1 hour at 37°C and 5 min at 95°C. First strand cDNA samples were stored at −20°C.

To measure the transcript levels of selected candidate genes by RT-qPCR, a 2.5× PCR reaction mix for qPCR (Syntol, Russia) was applied and analysis was performed on a 96-well DT-prime detection system (DNA-Technology, Russia). Each reaction was performed in triplicates in a reaction volume of 25 μl in high-profile 96-well unskirted PCR plates (BioRad, USA). All reactions contained 1 μl of cDNA diluted in 10 times, 10 μl of 2.5× Master Mix containing 6.25 mM MgCl_2_, 2.5 μl of 25 mM MgCl_2_ (till final concentration in a reaction mix 5 mM), 0.6 μl of 10 μM of each primers, 0.6 μl of 10 μM of a probe and 9.7 μl of DNase/RNase-free water. The PCR program consists of an initial 10 min template denaturation step at 94°C for enzyme activation, followed by 50 cycles of 94°C for 20 sec, 64°C for 10 sec, and 72°C for 15 sec. Nontemplate controls were also performed in triplicate for each gene set of primer pair and probe. Sets for all 18 candidate genes were analyzed in the same run. Each run was repeated three times. All PCR-mixes were pipetted by the automated pipetting system epMotion 5075 (Eppendorf, Germany). To verify the presence of genomic DNA contamination in the isolated total RNA, RT-qPCR were performed on equivalent amounts of total RNA without reverse transcription (as template) for each tumor sample.

Baseline and threshold values were automatically determined for all reactions in the plate using RealTime PCR v.7.3 software (DNA-Technology, Russia).

All RT-qPCR experiment data comply with the Minimum Information for Publication of Quantitative Real-Time PCR Experiments (MIQE) guidelines [32]. The MIQE checklist is presented in Supplementary Information (see Additional file 1).

### Analysis of gene expression stability by RT-qPCR

To analyze the stability of the candidate reference genes, four different methods were used [9]: comparative ΔCt method, geNorm, NormFinder and Haller equivalence test. Briefly, the geNorm tool is based on the principle that the expression ratio of two ideal reference genes should be constant in samples from different experimental conditions or cell types [12]. The expression stability value (M^geNorm^) of a gene is defined as the standard deviation of the log-transformed expression ratio for the particular gene relative to all other genes under investigation in a panel of cDNA samples. Lower values of M^geNorm^ correspond to higher gene expression stability. Because a freely available geNorm program does not allow the data to be processed efficiently if the RT-qPCR for each cDNA is repeated several times, we performed the geNorm analysis of our RT-qPCR data manually as described in [9].

The comparative ΔCt method compares a Ct ratio of “pairs of genes” within each sample and assigns a relative variation value M^ΔCt^ to each gene. Higher values of relative variation M^ΔCt^ correspond to lower gene expression stability.

NormFinder is a freely available program [46] which calculates the stability index using analysis of variance (ANOVA) on log-transformed expression values [1,30]. The NormFinder algorithm estimates overall gene expression variation M^NormFinder^ as well as the variation between subgroups, such as ER-positive and ER-negative breast cancer tissue samples. Higher values of M^NormFinder^ correspond to higher gene expression variability.

Haller equivalence test gives for each pair of samples *k, l* an α-confidence interval $\left[\delta_{\mathrm{kl},}^{L}\delta_{\mathrm{kl}}^{U}\right]$ for gene expression difference in samples *k, l*[31]. An integral measure of gene variability in Haller equivalence test approach (M^Haller^) was set to the 95-th percentile of a set ${\left\{\left|\delta_{\mathrm{kl}}^{L}\right|,\left|\delta_{\mathrm{kl}}^{U}\right|\right\}}_{k\neq l}$ with a confidence level α set to 0.05. Similarly to the other approaches considered in our study, the higher M^Haller^ value corresponds to higher gene expression variability. The analysis of gene expression stability using Haller equivalence test is described in details in [9].

Gene expression levels were estimated using a standard exponential model *A* = *I*/*E*^*Ct*^, where *A* is an expression level, *I* is a threshold value, *Ct* is a number of cycle corresponding to the threshold value, and *E* is a qPCR efficiency for a gene.

### Data generation for “quasi-genes”

In order to compare the stability of a set of reference genes with the stability of individual genes using standard methods that were used for the stability evaluation of genes (comparative ∆Ct method, geNorm, NormFinder, Haller equivalence test) we implemented a simple procedure that generates data for a “quasi-gene” that can be associated with a set of reference genes.

An expression level, assigned to a “quasi-gene” by this procedure, was equal to a normalization factor defined by the set of genes, or, in other words, to the geometric mean of expression levels for genes that belong to the set: $\tilde{A}=\sqrt[{n}]{\prod_{i=1}^{n}A_{i}}$,

In order to find an appropriate Ct-value $\tilde{C}$, required by some methods for stability evaluation, we used a standard exponential model *I* = *AE*^*Ct*^, where *A* is an expression level, *E* is a gene efficiency, *I* is a threshold, *Ct* is threshold cycle. We also accepted as a fact that all *Ct*-values for a fixed sample correspond to the same threshold $\tilde{I}$, and set an efficiency for a “quasi-gene” to the geometric mean of efficiency values of genes in the set $\left(\tilde{E}=\sqrt[{n}]{\prod_{i=1}^{n}E_{i}}\right)$. Due to the exponential model assumptions, $\log_{\tilde{E}}\tilde{I}=\log_{\tilde{E}}\tilde{A}+\tilde{C}\log_{\tilde{E}}\tilde{E}=\log_{\tilde{E}}\tilde{A}+\tilde{C}$, so Ct-value, assigned to by the procedure to a “quasi-gene” for a fixed sample, was equal to

$$\log_{\tilde{E}}\tilde{I}-\log_{\tilde{E}}\tilde{A}=\log_{\tilde{E}}\tilde{I}-\log_{\tilde{E}}\sqrt[{n}]{\prod_{i=1}^{n}A_{i}}=\log_{\tilde{E}}\tilde{I}-\frac{1}{n}\sum_{i=1}^{n}\log_{\tilde{E}}\frac{\tilde{I}}{E_{i}^{Ct_{i}}}=\frac{1}{n}\sum_{i=1}^{n}Ct_{i}\log_{\tilde{E}}E_{i}.$$

The RT-qPCR data contained 3 replicates for every gene and 3 replicates for every sample, and hence, there were nine Ct-values for each gene-sample pair. Taking the respective Ct-values of this 3x3 matrix for each gene of a system we got the corresponding matrix of Ct-values for a “quasi-gene”.

## Results

The strategy for the selection of reference genes that was used in this study consists of the following stages: the large-scale screening of an extended list of candidate reference genes based on microarray data series, the selection of a candidate gene set for validation by RT-qPCR, and the determination of the final set of reference genes.

### Large-scale selection of an extended list of candidate reference genes based on microarray data

The large-scale selection of the extended list of candidate reference genes was performed by a selection of genes that are present in sub-lists of most stable genes in all four analyzed microarray data sets (see the details in the Methods). Note that this approach allows a combining of microarray studies that utilize different microarray technologies. The total number of such genes was 25 (Table 3). Sixty percent of these genes (15 genes) were present in the list of maintenance genes expressed across eleven human adult and fetal tissues [11], and 9 percent (3 genes) were also present in the list of 47 transcripts expressed at the same level across eleven human adult and fetal tissues [11]. The extended list of candidates included eight *RPL* and eight *RPS* genes. These results are concordant with the results of [14,15,24].

**Table 3:** An extended list of candidate reference genes

**Gene symbol**	L11*	L11_eq**	L19***
*ACTG1*	+	–	+
*EEF1A1*	+	+	+
*HCFC1*	–	–	–
*HNRNPA1*	–	–	–
*HUWE1*	–	–	–
*NACAP1*	–	–	–
*PKD2L1*	–	–	–
*PTMA*	+	–	+
*RPL10*	+	–	–
*RPL22*	–	–	–
*RPL23A*	–	–	–
*RPL37A*	+	+	+
*RPL39*	+	–	+
*RPL41*	+	+	+
*RPL7*	+	–	+
*RPL9*	+	–	+
*RPS10*	+	–	+
*RPS15A*	–	–	–
*RPS16*	+	–	+
*RPS18*	+	–	+
*RPS20*	+	–	–
*RPS23*	–	–	+
*RPS3A*	–	–	+
*RPS4X*	+	–	–
*TPT1*	+	–	+

*L11: presence of a gene in the list of maintenance genes expressed in eleven human adult and fetal tissues [11].

**L11_eq: presence of a gene in the list of 47 transcripts expressed at the same level in eleven human adult and fetal tissues [11].

***L19: presence of a gene in the list of 451 housekeeping genes from 19 normal human tissue types [15].

Such genes as *ACTB* and *GAPDH*, which are widely used for normalization of RT-qPCR data, were not included into the resulting extended list of candidate genes. The *GAPDH* gene did not belong to any list of top-500 stable probesets, while gene *ACTB* (as well as *UBC*) belonged to three lists of top-500 stable probesets, but was not present in top-500 list corresponding to a dataset of histologically normal epithelium from breast cancer patients and cancer-free prophylactic mastectomy patients (GSE20437). It is also worth noting that *ACTB* and *UBC* (in contrast with, e.g., *GUSB*) had high intergroup difference in expression level for samples from cancer and cancer-free patients. However, the intragroup variation was low for each of the groups. Hence, the assessment of the expression level of these genes can be potentially used for the estimation of a tumor/normal epithelium ratio in a biopsy sample. An ability of housekeeping genes to define different biological states was also reported in [15].

### Selection of a gene set for the experimental validation and RT-qPCR validation

The selection of a gene set for validation from the preliminary extended list was performed by an identification of genes with the highest stability values in the majority of the analyzed microarray data series, but the following additional limitation was imposed: a simultaneous selection of genes with known interactions, as well as a selection of genes with similar biological functions should be minimized. Informally, this limitation increases the independence of genes in the selected set.

Nine genes from the extended list of candidate reference genes (*TPT1*, *HCFC1*, *PTMA*, *RPL23A*, *RPL37A, RPL39, RPS23, HUWE1, EEF1A1*) were selected for the experimental validation of the expression stability and comparison with other genes traditionally used as reference genes in RT-qPCR studies of breast tissue (*ACTB*, *GAPDH*, *RPLP0*, *GUSB*, *TFRC*, *SF3A1*, *MRPL19*, *PSMC4*, *TBP*[2,23,27,47,48]) (Table 1). As no unique criterion exists for the comparison of gene expression stability, we combined four different approaches: comparative ΔCt method [8], geNorm [12], NormFinder [1,30], and Haller equivalence test [31] (see Methods section for the details).

**Figure 1:**
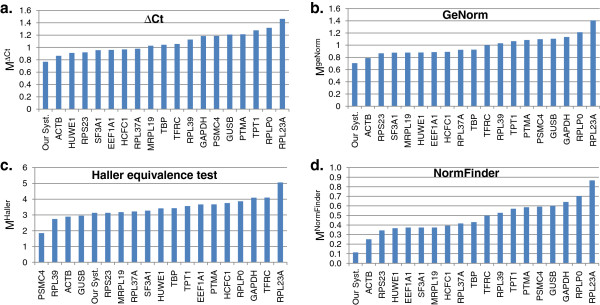
**Evaluation of candidate reference gene expression stability by RT-qPCR.** Gene expression variations calculated for aggregated set of samples by **a:** comparative ΔCt (M^ΔCt^); **b:** geNorm (M^geNorm^) and **d:** NormFinder (M^NormFinder^). **c:** Maximum fold changes of gene expression levels M^Haller^ calculated by Haller equivalence test.

The simplest method, the comparative ΔCt method, uses only raw values of threshold cycles Ct. An application of the ΔCt method to the results of RT-qPCR analysis of 39 breast cancer tissue samples for 18 genes from the set for validation gave the following results (Figure 1a, Additional file 6: Figure S1). Ten genes, namely, *ACTB*, *HUWE1*, *RPS23*, *EEF1A1*, *SF3A1*, *HCFC1*, *RPL37A*, *MRPL19*, *TBP* and *TFRC* showed nearly the same expression stability for the whole set of samples (Figure 1a). The *RPL23A* gene displayed the lowest stability in the whole set of samples and ER-negative tumors (Additional file 6: Figure S1). The highest variability for ER- positive tumors was exhibited by *PSMC4*.

In contrast to the comparative ΔCt method, geNorm, NormFinder and Haller equivalence test use expression levels as input data. The values of expression stability M^geNorm^ for 18 candidate genes generated by the most widely used algorithm geNorm are presented in Figure 1b and Additional file 6: Figure S1. The 10 top-ranked genes were similar to the top-10 genes in the rating generated by the comparative ΔCt method in the whole set of samples: stability values of their expression were nearly the same in each sample group and only the exact order of genes was slightly different.

Unlike comparative ΔCt method and geNorm, NormFinder and Haller equivalence test examine the expression stability of each candidate independently from other candidates. The results of NormFinder application to the studied set of genes is presented in Figure 1d and Additional file 6: Figure S1. In intergroup variation analysis groups were formed by ER-positive and ER-negative samples, respectively (Additional file 7: Figure S2). Higher stability was observed for *ACTB, RPS23, HUWE1, EEF1A1, MRPL19, SF3A1, HCFC1, RPL37A* and *TBP*.

The Haller equivalence test allows to evaluate a fold change value (M^Haller^) of candidate reference genes expression level between different samples for a given significance value. The results of the expression variability estimation for the 18 candidate genes studied are presented in Figure 1c and Additional file 6: Figure S1. In contrast to the comparative ΔCt method, geNorm and NormFinder *PSMC4* demonstrated the lowest variability for the whole set of samples as well as for ER-negative and ER-positive tumors. The next gene in the stability ranking was *RPL39*, the third gene was *ACTB* that demonstrated lowest variation according to other methods of stability evaluation that A large group of genes from the validation set (*GUSB, RPS23, MRPL19, RPL37A, SF3A1, HUWE1, TBP*) showed very similar M^Haller^ values and were the next in stability ranking. In concordance with the results of the comparative ΔCt method, geNorm and NormFinder, *RPL23A* had the greatest expression variability. The relatively high M^Haller^ values of all candidate genes are consistent with highly heterogeneous nature of biopsies and with other studies [15,47]. Also it is worth noting that M^Haller^ stability values are “pessimistic” in comparison with other methods as they represent the “poor case”, but not the “averaged” one (see details in Methods).

An important inference is that such genes as *GAPDH* and *RPLP0*, commonly employed as reference genes (e.g., in the commercial Oncotype DX diagnostic test) were ranked among the most variable candidate genes by almost all methods.

### Final selection of reference genes

Thus four rankings of gene expression stability were obtained by the following methods: comparative ∆Ct method [8], geNorm [12], NormFinder [1,30] and Haller equivalence test [31]. In spite of the fact that generally the results of all stability evaluation methods are consistent each of these ranking has its own order of the genes. Based on both four stability rankings and intergroup variations calculated by NormFinder we formed a subset of the best 5 reference genes: *ACTB, RPS23, HUWE1, EEF1A1, SF3A1* (other good choices can be obtained by a replacement of *EEF1A1* or *SF3A1* by *MRPL19*). Note that three out of five genes in this set (*RPS23*, *HUWE1*, *EEF1A1*) were selected by microarray analysis and were top-ranked in the extended list of candidate genes. *ACTB* was not included in the extended list of candidate genes only because it had high intergroup variation between normal epithelium samples from cancer and cancer-free patients. However it showed low variability value for other microarray data series used for the selection of the extended list.

In order to validate the selected subset of reference genes we added a “quasi-gene” corresponding to the set of 5selected genes (see *Methods* section for details) and calculated the stability measures by all four methods (Table 4). The results show that the selected subset clearly outperforms not only every individual gene from the tested set, but also a system of reference genes used by OncotypeDX test. The only method that did not rank the selected set as the most stable was the Haller equivalence test: it ranked the individual gene *PSMC4* higher than “quasi-genes”.

**Table 4:** Stability characteristics evaluated for the system of 18 individual genes and 2 “quasi-genes” – one corresponding to the selected system of reference genes, and the other corresponding to the system of reference genes used in OcotypeDX test

	**Aggregate set of samples**			
	**ΔCt**	**geNorm**	**Haller**	**Norm Finder**
Best gene*	*ACTB*	*ACTB*	*PSMC4*	*ACTB*
	0.864	0.789	1.853	0.252
Selected subset	0.767	0.706	3.138	0.114
OncotypeDX subset	0.866	0.805	3.157	0.263

*Best gene corresponds to gene with the least variability characteristic among 18 genes.

## Discussion

RT-qPCR has become the most popular method for detection and quantification of mRNA transcripts. An efficient normalization enables the gathering of reproducible and biologically relevant RT-qPCR data correcting non-biological sample-to-sample variations that could be introduced by protocol-dependent inconsistencies [1,30]. To achieve an efficient normalization the use of internal control genes (reference genes) has been established as the gold standard normalization method. Formerly, the genes that have housekeeping roles in basal cellular activities were selected as internal control for gene expression studies. However, it is obvious now that a housekeeping function does not guarantee that a gene has a stable expression level. It was well documented that expression levels of many commonly used housekeeping genes, including *GAPDH*, *ACTB*, *RPS18*, *UBC*, *B2M*, *HPRT1* and others, may vary significantly in different tissues and cells [4,7,9,11-15]. Therefore it is necessary to identify novel candidate reference genes for given individual studies.

Our goal was to select a set of reference genes that can be efficiently used for both research and clinical analysis of breast cancer samples with an unknown hormone receptor status and different cancer status. In order to achieve this goal we developed the strategy for the selection of reference genes that consists of the following stages: 1) selection of an extended list of candidate reference genes based on microarray data, 2) selection of a set of candidate genes for validation by RT-qPCR and 3) selection of the final set.

Similarly to the studies presented in [14,24] we started from the analysis of available microarray datasets, however, we used variation characteristics that are more stable with respect to outliers. This analysis allowed us to identify 25 genes with stable expression level for the studied sample types (Table 3). Forming the list of candidate genes for validation, we took into account the possible interactions of genes and their involvement in the same biological pathways. The final set of reference genes was selected based on four stability rankings generated by the most widely used methods for evaluation of gene expression variability. This allowed us to take advantage of all considered methods. We noted that both microarray and RT-qPCR data allows to evaluate only the stability of a transcript portion of an individual gene in a fixed amount of total RNA, or, in other words, to evaluate the relative stability of gene expression. Hence it is a natural idea to use tools that estimate the expression stability of a gene relative to the expression of other genes, e.g. the comparative ∆Ct method [8] and the geNorm [12]. However, low values of such relative variation characteristics can be a result of gene co-regulation. That is why the usage of tools based on stability measures of individual genes independently on other genes such as NormFinder and Haller equivalence test is very reasonable.

All methods used for the gene stability evaluation showed that the genes selected by microarray analysis are comparable with or even better than traditionally used reference genes: in the final subset 3 out of 5 genes belonged to the list of genes selected by means of microarray analysis. These three genes are *RPS23*, *HUWE1* and *EEF1A1*.

## Conclusions

We presented experimentally validated evidence that a selection of reference genes for RT-qPCR can be efficiently performed by combining a preliminary search based on the high-throughput analysis of microarray datasets with subsequent selection and validation using RT-qPCR and simultaneous examination of different expression stability measures. We showed that the identified set of reference genes, including *ACTB, RPS23, HUWE1, EEF1A1* and *SF3A1* proved to be less variable and thus potentially more efficient for research and clinical analysis of breast samples in comparison with individual genes and a set of reference genes used in OncotypeDX assay.

## Abbreviations

RT-qPCR: Reverse transcription quantitative real-time polymerase chain reaction; MIQE: Minimum Information for Publication of Quantitative Real-Time PCR Experiments; ER: Estrogen receptor; PR: Progesterone receptor; RQI: RNA quality indicator; IHC: immunohistochemistry.

## Competing interests

The authors declare that they have no competing interests.

## Authors’ contributions

DVM contributed to the conception of the study, assisted in the RT-qPCR studies and data processing, wrote the first draft of the manuscript. NAK performed the RNA sample preparations, part of RT-qPCR studies, contributed to the data processing and interpretation and helped to draft the manuscript. EOM performed the majority of RT-qPCR studies. NNF performed the majority of RT-qPCR data processing and helped to draft the manuscript. AEL performed the processing of microarray data. MUS performed the processing of the patient data and some of immunohistochemistry analysis of biopsies. VVG contributed to study conception, processing of the RT-qPCR and microarray data, data interpretation and edited the manuscript. US and AGT contributed to study conception, and critical manuscript review. All authors read and approved the final manuscript.

## Supplementary Material

Additional file 1 MIQE Click here for file

Additional file 2 Clinical information of tumor samples. Click here for file

Additional file 3 qPCR target information. Click here for file

Additional file 4 qPCR efficiency. Click here for file

Additional file 5 Calibration curves for efficiency. Click here for file

Additional file 6: Figure S1 Evaluation of candidate reference gene expression stability by RT-qPCR. Click here for file

Additional file 7: Figure S2 Intergroup variation characteristic of candidate reference genes calculated by NormFinder. Click here for file
