# Supplementary figures and images for: High-throughput identification of reference genes for research and clinical RT-qPCR analysis of breast cancer samples
Source: J Clin Bioinforma. 2013 Jul 22;3:13. doi: 10.1186/2043-9113-3-13 (PMC3726509; doi:10.1186/2043-9113-3-13)

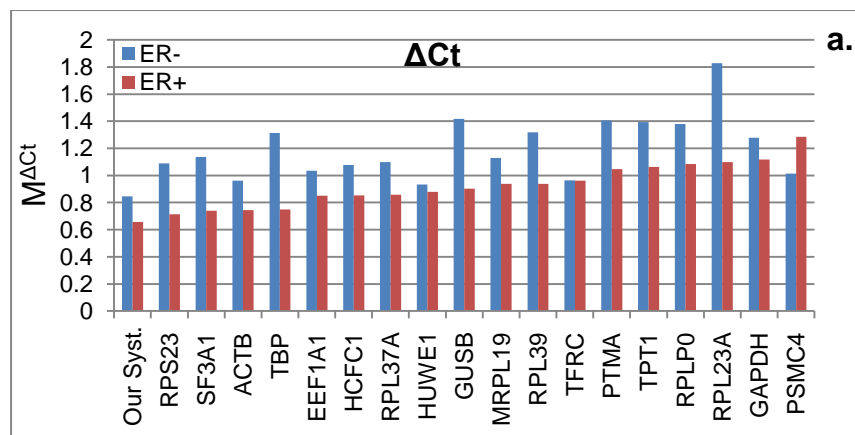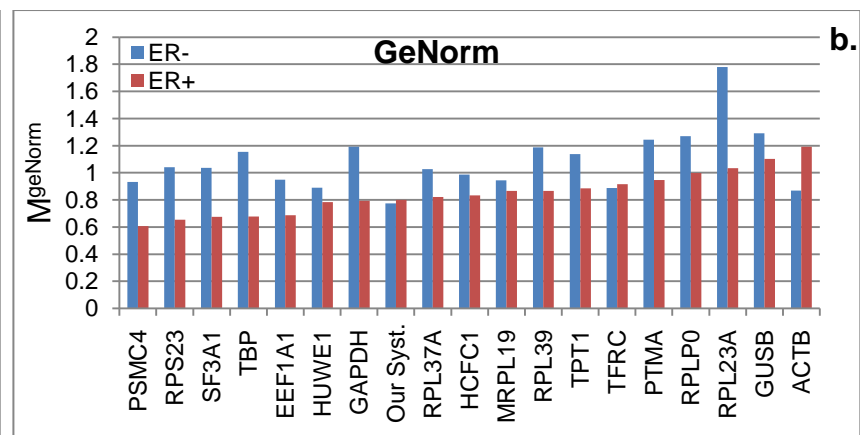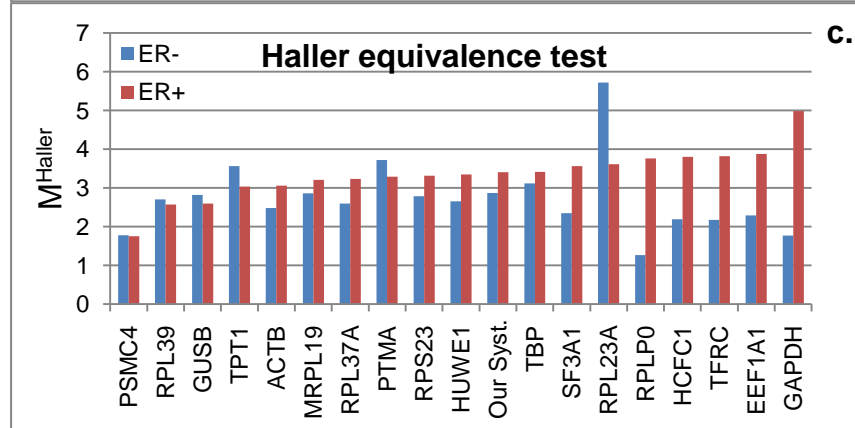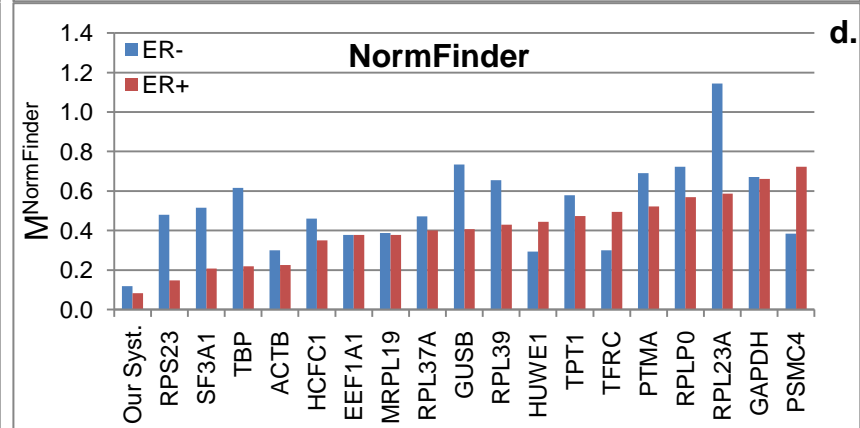

Supplement: Additional file 6: Figure S1 — Evaluation of candidate reference gene expression stability by RT-qPCR. [file 2043-9113-3-13-S6.pdf]
