# Supplementary material for: High-throughput identification of reference genes for research and clinical RT-qPCR analysis of breast cancer samples
Source: J Clin Bioinforma. 2013 Jul 22;3:13. doi: 10.1186/2043-9113-3-13 (PMC3726509; doi:10.1186/2043-9113-3-13)
Supplement: Additional file 7: Figure S2 — Intergroup variation characteristic of candidate reference genes calculated by NormFinder. [file 2043-9113-3-13-S7.pdf]

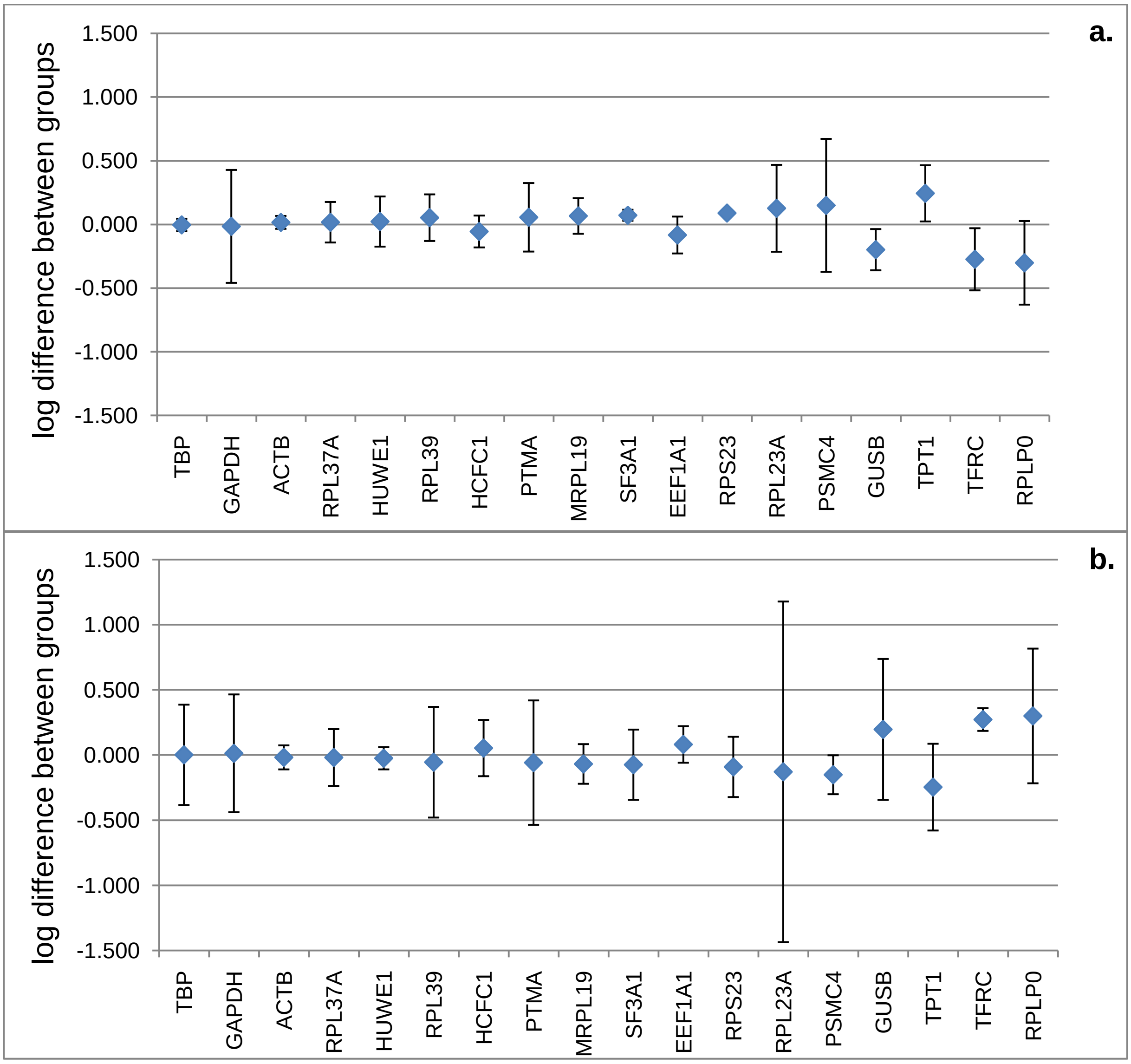

Figure S2 Candidates ranked by the NormFinder's model-based and pairwise comparison approaches. Using the ER+ and ER- groups of data, the intergroup variation is plotted (*blue squares*) as the expression difference between the two groups. The intragroup variation is indicated by *vertical bars* (a: ER+ samples; b: ER- samples) that give a confidence interval for the difference. Because the average of the intergroup variations is always 0, in the case of chart a an intergroup variation of >0 implies that the gene shows systematically higher expression in ER+ tumors than in ER- tumors and the opposite if the value is <0. Intergroup variations on charts a and b are mirror symmetric with respect to zero level.
